# Type2 diabetes mellitus prediction using data mining algorithms based on the long-noncoding RNAs expression: a comparison of four data mining approaches

**DOI:** 10.1186/s12859-020-03719-8

**Published:** 2020-08-27

**Authors:** Faranak Kazerouni, Azadeh Bayani, Farkhondeh Asadi, Leyla Saeidi, Nasrin Parvizi, Zahra Mansoori

**Affiliations:** 1 Department of Laboratory Medicine, School of Allied Medical Sciences, Shahid Beheshti University of Medical Sciences, Tehran, Iran; 2 Department of Health Information Technology and Management, School of Allied Medical Sciences, Shahid Beheshti University of Medical Sciences, Tehran, Iran; 3 Department of Clinical Biochemistry, School of Medicine, Tehran University of Medical Sciences, Tehran, Iran; 4 Department of Genetics, Faculty of Medicine, Babol University of Medical Sciences, Babol, Iran

**Keywords:** Data mining, Gene expression, Machine learning algorithms, Type 2 diabetes mellitus

## Abstract

**Background:**

About 90% of patients who have diabetes suffer from Type 2 DM (T2DM). Many studies suggest using the significant role of lncRNAs to improve the diagnosis of T2DM. Machine learning and Data Mining techniques are tools that can improve the analysis and interpretation or extraction of knowledge from the data. These techniques may enhance the prognosis and diagnosis associated with reducing diseases such as T2DM. We applied four classification models, including K-nearest neighbor (KNN), support vector machine (SVM), logistic regression, and artificial neural networks (ANN) for diagnosing T2DM, and we compared the diagnostic power of these algorithms with each other. We performed the algorithms on six LncRNA variables (LINC00523, LINC00995, HCG27_201, TPT1-AS1, LY86-AS1, DKFZP) and demographic data.

**Results:**

To select the best performance, we considered the AUC, sensitivity, specificity, plotted the ROC curve, and showed the average curve and range. The mean AUC for the KNN algorithm was 91% with 0.09 standard deviation (SD); the mean sensitivity and specificity were 96 and 85%, respectively. After applying the SVM algorithm, the mean AUC obtained 95% after stratified 10-fold cross-validation, and the SD obtained 0.05. The mean sensitivity and specificity were 95 and 86%, respectively. The mean AUC for ANN and the SD were 93% and 0.03, also the mean sensitivity and specificity were 78 and 85%. At last, for the logistic regression algorithm, our results showed 95% of mean AUC, and the SD of 0.05, the mean sensitivity and specificity were 92 and 85%, respectively. According to the ROCs, the Logistic Regression and SVM had a better area under the curve compared to the others.

**Conclusion:**

We aimed to find the best data mining approach for the prediction of T2DM using six lncRNA expression. According to the finding, the maximum AUC dedicated to SVM and logistic regression, among others, KNN and ANN also had the high mean AUC and small standard deviations of AUC scores among the approaches, KNN had the highest mean sensitivity and the highest specificity belonged to SVM. This study’s result could improve our knowledge about the early detection and diagnosis of T2DM using the lncRNAs as biomarkers.

## Background

Diabetes mellitus (DM) is one of the most prevalent chronic non-communicable diseases (NCD) around the world; about 90% of the patients who have diabetes suffer from Type 2 DM (T2DM) [1]. The risk of developing T2DM is strongly associated with many predispositions, behavioral, and environmental risk factors and also genetic factors [1–4]. Besides the genetic factors, strong evidence indicates that factors such as obesity and physical inactivity are the main nongenetic determinants of the disease [5, 6]. T2DM can range from predominant insulin resistance with relative insulin deficiency to dominant defective secretion with insulin resistance [4]. It is often related to metabolic syndrome problems. Individuals who have impaired glucose tolerance are high-risk subjects of type 2 diabetes [6].

Studies demonstrate a drastic increase of the disease in recent decades. The trends estimate that by 2035, more than 520 million people will be affected by the disease [7]. People who suffer from T2DM are susceptible to many forms of complications leading to morbidity and mortality in these patients. Many studies emphasize the genetic factors in the pathogenesis of T2DM [3, 8, 9]. Long non-coding RNAs (long ncRNAs, lncRNA) are subsets of RNA, specified as being transcripts with lengths exceeding 200 nucleotides that could not be translated into protein [10]. Long non-coding RNAs (lncRNAs) belong to a heterogeneous class of regulatory lncRNAs with transcript lengths > 200 nucleotides, which have a positive role in the development and growth of several various diseases including T2DM supporting the hypothesis that abnormal expression of LncRNAs is related to various diseases [11]. Besides, considering the significant role of lncRNAs in disease pathogenesis, increasing researches suggest using them to improve diagnosis, prognosis, and clinical management of T2DM. Genome-wide association studies (GWAS) have recently introduced several particular diabetes-related loci in the human genome [3]. Also, many studies discovered the relationship between more than 100 susceptible loci and T2DM at a genome-wide significant level [3, 8, 12]. Deregulation of genes located in GWAS defined loci may be risk factors for human diseases concerning which we applied the GWAS catalog to select six lncRNAs (LINC00523, LINC00995, CG27_201, TPT1-AS1,LY86-AS1, DKFZP) as our gene targets for the present study [3]. Knowledge Discovery in Databases (KDD) or data mining are techniques for the computational process of discovering patterns in large datasets containing various approaches such as artificial intelligence, machine learning, statistics, and database systems [13]. These methods are applied to recognize patterns in data, prediction, association, and classification problems [1, 2, 8, 13]. Considering the importance of early detection of T2DM, machine learning and Data Mining techniques are tools that can improve the analysis and interpretation or extraction of knowledge from the data [14, 15]. These techniques may enhance the prognosis and diagnosis associated with life quality, reducing diseases such as T2DM [15, 16].

To date, several other studies tried to predict diabetes mellitus using outstanding data mining techniques [17–19]. Vijayan et al. [20] applied the expectation-maximization algorithm, KNN algorithm, K-means algorithm, amalgam KNN algorithm, and ANFIS algorithm to predict and diagnose Diabetes Mellitus. They used the UCI dataset containing blood test and demographic variables, and their results showed that EM possessed the least classification accuracy and amalgam KNN, and ANFIS provided better classification accuracy of more than 80 and 80%, respectively. Another study conducted by Saravananathan et al. [21] used popular classification algorithms, including J48, Support Vector Machines (SVM) Classification and Regression, Tree CART, and k-Nearest Neighbor (kNN) for diabetic data. Their performance indicators were accuracy, specificity, sensitivity, precision, error rate. They found that the J48 technique’s performance was remarkably superior to the other three techniques for the classification of diabetes data. Meng et al. [18] compared three data mining models of logistic regression, ANN, and decision tree for predicting diabetes mellitus or prediabetes by risk factors. They gathered information about demographic characteristics, family diabetes history, anthropometric measurements, and lifestyle risk. The decision tree model (C5.0) had the best classification performance with an accuracy of 77.87% with a sensitivity of 80.68% and specificity of 75.13%. Another study performed by Saeidi et al. [3] used logistic regression to assess the diagnostic value of LY86-AS1 and HCG27_201 as biomarkers for T2DM. They obtained a sensitivity of 64.6%, and specificity of 79.8%. Another study [2] used two other lncRNAs, including LINC00523 and LINC00994 expressions, for the evaluation of their potential diagnostic value for T2DM. They applied logistic regression and achieved a sensitivity of 81.44% and specificity of 61.11%. In our study, we combined six lncRNAs as variables for the first time and applied four classification models, including classification algorithms like K-nearest neighbor (KNN), support vector machine (SVM), logistic regression, and artificial neural networks (ANN) for diagnosing T2DM, and we compared the diagnostic power of these algorithms with each other. In the present study, we aimed to find the best data mining approach for the prediction of T2DM using six lncRNA expression. The result of this study could improve our knowledge about the early detection and diagnosis of T2DM using the lncRNAs as biomarkers [22].

## Methods

The primary aim of the present study was to implement four models to predict DT2M applying data mining techniques based on the lncRNA variables. The research objectives of our study were:

1. Implementing data mining techniques for prediction of the DT2M.
2. Comparing the applied methods.
3. selecting the best model for the T2DM prediction.

We used the variables for predicting T2DM and comparing the performance of the various data mining techniques. For the implementation of the algorithms, we used ANACONDA3–5.2.0 64 bit a free and open-source platform distribution of python programming language with a vast number of modules, packages, and rich libraries that provide various methods for classification problems. For obtaining the best amount of performance in the models, 10-fold cross-validation performed on the dataset. In dealing with the small data sets, cross-validation is a prominent strategy for estimating the performance. Cross-Validation is a performance evaluation technique commonly used in practice. Here, the data set is repeatedly partitioned into two non-overlapping parts, a training set, and a hold-out set. For each partitioning, the hold-out set is used for testing, while the remainder is used for training. The two most popular variants are ten-fold cross-validation (10-fold CV), where the data is split into ten mutually disjoint folds [23].

Since our samples were more than 100, and to be sure that each fold contains the same proportion of healthy and diabetic individuals, we used the stratified 10-fold cross-validation approach [24]. Therefore, the results are reliable and more credible.

We applied four popular data mining approaches on the lncRNA variables, regression, k-nearest neighbors, SVM, and neural network classification algorithms.

### KNN algorithm

The k-nearest neighbor’s algorithm (k-NN) is an algorithm for classifying variables regarding the closest training data in the feature space. K-NN uses an instance-based learning method, which is one of the simplest algorithms among data mining techniques. This method considers the nearest neighbors to each object and decides to dedicate the object to classes [22, 25].

### SVM algorithm

Support Vector Machine (SVM) is a supervised algorithm which divides the feature space called hyperplanes considering the target classes. SVM computes classification by maximizing the margin of the hyperplane that intercepts classes. This algorithm plots a multidimensional hyperplane that divides classes and increases the margin between classes to enhance the accuracy of classification. We used different kernel functions embedded in the SVM class of SVC library in python framework as a quadratic, polynomial, radial basis, etc. to classify the instance and to detect the best accuracy among them [25–27].

### Artificial neural network

**Fig. 1:**
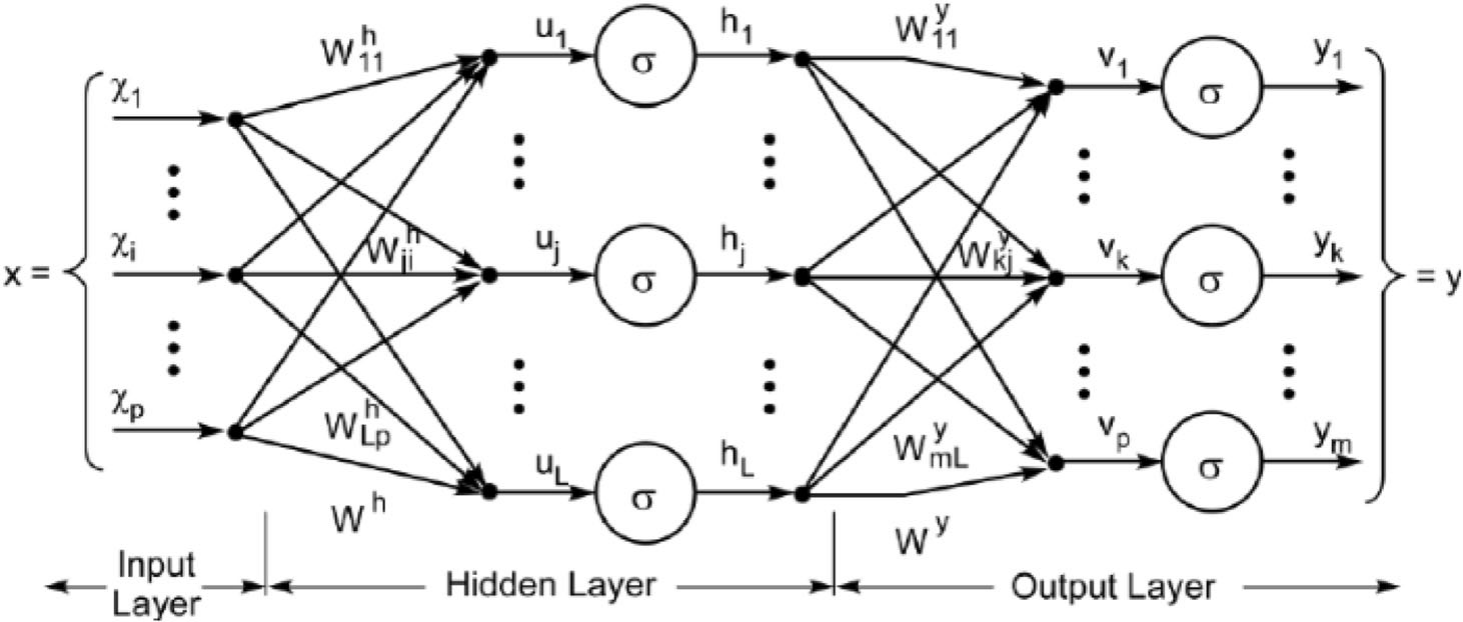
Artificial Neural Network structure

Artificial Neural Network is a data processing algorithm that simulates the biological neural network in its computations. A common problem in using ANN is that they act fundamentally as a black box and the parameters are set by the model so we cannot demonstrate them [28], we can just apply the model in our problems and obtain the high performance. We used Multilayer Perceptron Neural Networks (MLPNN). The structure of a multi-layer perceptron neural network has been demonstrated in Fig. 1. It maps a set of input data into a set of appropriate output classes. It includes three layers input layer, hidden layer & output layer. The principal function of neurons of the input layer is to divide input Xi into neurons in the hidden layer. The neuron of the hidden layer adds the appropriate weights of Wij to the input variables. The output formula is:

$$ \mathrm{Yj}=\mathrm{f}\ \left(\sum \mathrm{Wji}\ \mathrm{Xi}\ \right) $$

**Table 1:** The lncRNAs as inputs of algorithms

number	Variables
LncRNA Variables	
1	LINC00523
2	LINC00995
3	HCG27_201
4	TPT1-AS1
5	LY86-AS1
6	DKFZP
Demographic Variables	
7	Sex
8	Age
9	Weight
10	Height
11	BMI
12	FBS

**Fig. 2:**
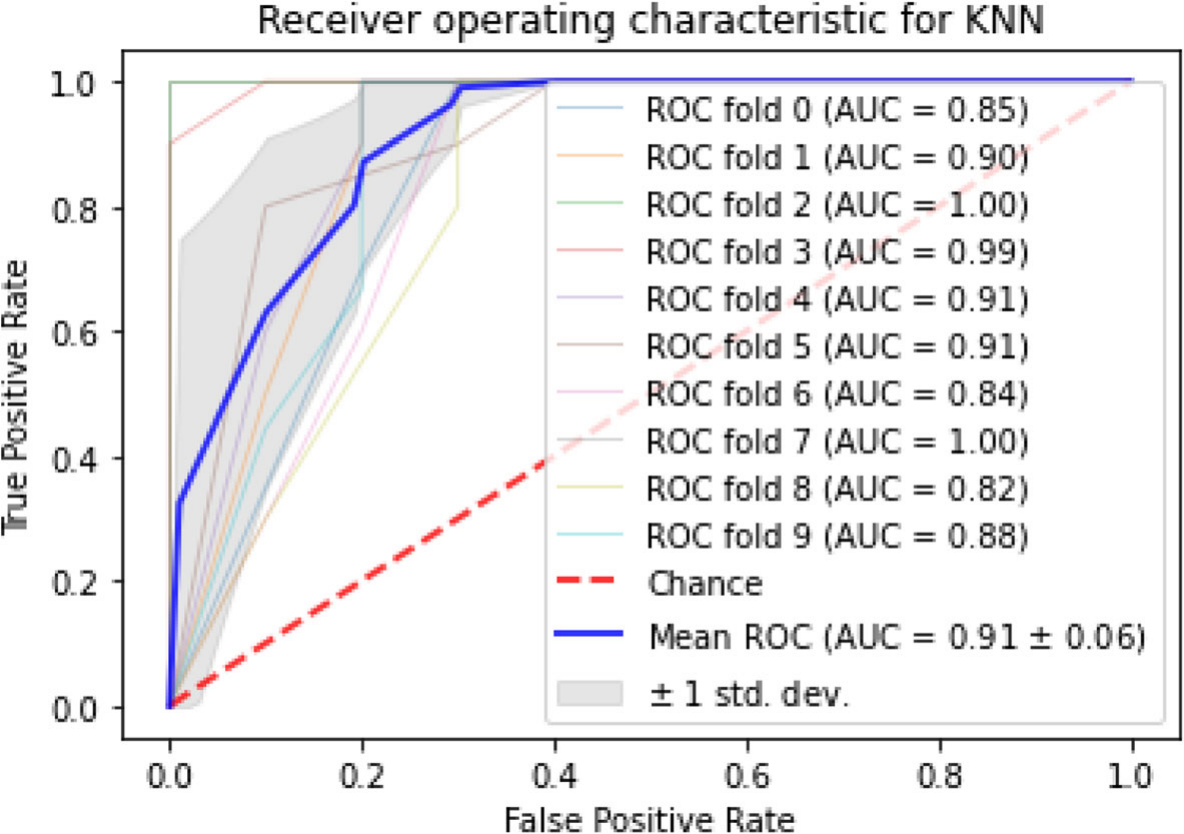
The ROC for KNN

Where f is a simple threshold function that we considered sigmoid and hyperbolic tangent function [25].

In the present study, a Multi-layer Perceptron Neural Networks (MLPNN) was performed. The structure of MLPNN is as shown in Fig. 1. It makes a map of input data onto a set of suitable output data.

**Fig. 3:**
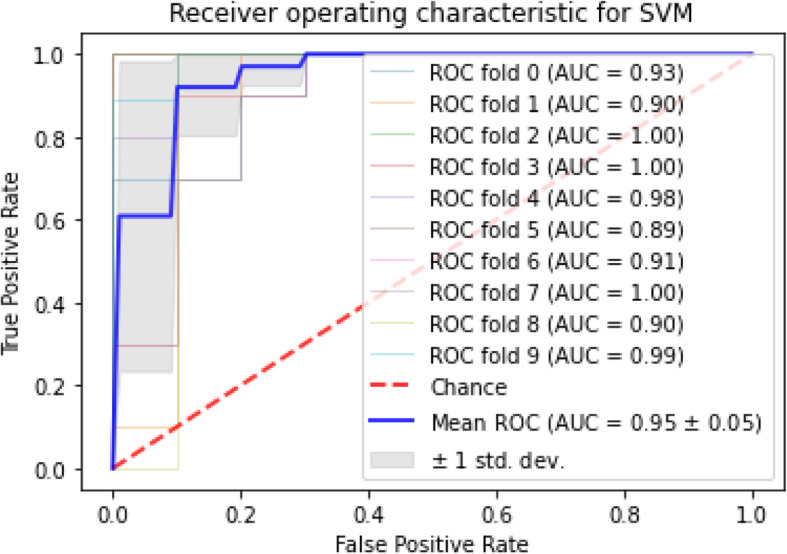
The ROC for SVM

The RBF networks are another type of neural network. In MLP, each neuron considers the weighted sum of its input values, in which each input value is multiplied by a coefficient, and the results are the sum of values. RBF is a more intuitive approach to MLP. An RBFN classifies the inputs by calculating the input’s similarity to examples from the training set. Each RBFN neuron stores one of the examples from the training set as a “prototype.” for classification of new input, in each neuron, the Euclidean distance between the input and its prototype is calculated. The input is dedicated to a class when it has more similar to that class than the other classes.

### Logistic regression

Logistic regression is a common approach for predictive modeling practices. The function p(X) provides probability output between 0 and 1 for all values of X, where X1–Xp are the predictors. The coefficients β0–βp are estimated using maximum likelihood estimation

$$ \mathrm{p}\left(\mathrm{X}\right)=\frac{{\mathrm{e}}^{\upbeta_0+{\upbeta}_1{\mathrm{X}}_1\kern0.5em +\cdots +{\upbeta}_{\mathrm{p}}{\mathrm{X}}_{\mathrm{p}}}}{1+{\mathrm{e}}^{\upbeta_0+{\upbeta}_1{\mathrm{X}}_1\kern0.5em +\cdots +{\upbeta}_{\mathrm{p}}{\mathrm{X}}_{\mathrm{p}}}} $$

### Dataset

This study was based on the data obtained from three previous research conducted by Saeidi et al. and Mansoori et al. [2, 3] and the research of Parvizi and colleagues, which is not published yet. We integrated these three studies, and our data mining analysis was implemented in their studies. The data were collected from 200 unrelated Iranian subjects, 100 T2DM patients, and 100 healthy individuals, matched for age and sex. T2DM patients were recruited from individuals who referred to the Diabetic Clinic at Shohada Hospital, Tehran, Iran. In the current study, we applied six lncRNAs expression and also six demographic variables, including sex, age, weight, height, BMI, and FBS for analysis and inputs of algorithms. For the preprocessing phase, we normalized the data inputs for KNN, SVM, and ANN models. We also had low missing variables, and we replaced them with zero (Table 1).

### lncRNA extraction and selection

Increasing evidence has suggested several lncRNAs are implicated in T2DM pathogenesis. Recently, human β-cell transcriptome analysis showed lncRNAs dynamic regulation and abnormal expression of lncRNAs in T2DM [29]. However, the extent of lncRNA deregulation in T2DM has yet to be determined. To date, more than100 susceptibility loci have been identified as being associated with T2DM at a genome-wide significant level [2, 30]. Considering this into account and by querying the GWAS catalog, we candidated 6 lncRNAs (LY86-AS1, HCG27_201, LINC00523, LINC00994, TPT1-AS1and DKFZP) as target genes for this study.

The large scale GWAS have recognized approximately 80 SNPs that were susceptible to T2DM [31]. From there, we used the GWAS catalog access in June 2017 to create a list of SNPs associated with T2DM. In the current study, we selected six lncRNA for expression analysis according to the scan carried out in the study of Mansoori et al. [2] and Saeedi et al. [3] We selected variants that had associations with increased risk of T2DM. We applied a quantitative PCR analysis of lncRNA expression levels in the 200 samples. We calculated the respective amount of each lncRNAs applying the 2-ΔΔct as means of duplicate measurements.

### Analysis and evaluation criteria

To select the best performance data mining algorithms in predicting diabetic patients, we considered AUC, sensitivity, specificity, and plotted ROC curve for the folds we ran and showed the average curve and its range [19, 26].

## Results

Table 2 shows the significant downregulation of PBMC expressions of the variables in the T2DM group compared with the control group. The AUC of each classification technique has been demonstrated in Table 3.

**Table 2:** Relative expression of the variables

Variables	Diabetes	Control	***p***-value
	∆CT ± SEM	∆CT ± SEM	
LINC00523	7.48 (6.96–8.00)	3.64 (3.10–4.18)	< 0.0001
LINC00995	6.97 (5.83–8.11)	5.82 (4.85–6.79)	0.44
HCG27_201	9.15 (8.46–9.84)	6.25 (5.56–6.94)	0.004
TPT1-AS1	5.30 (5.07–5.53)	3.28 (2.77–3.79)	< 0.0001
LY86-AS1	9.8 (9.93–10.67)	6.13 (5.39–6.87)	0.002
DKFZP	6.43 (5.68–7.18)	5.10 (4.53–5.67)	0.163

**Fig. 4:**
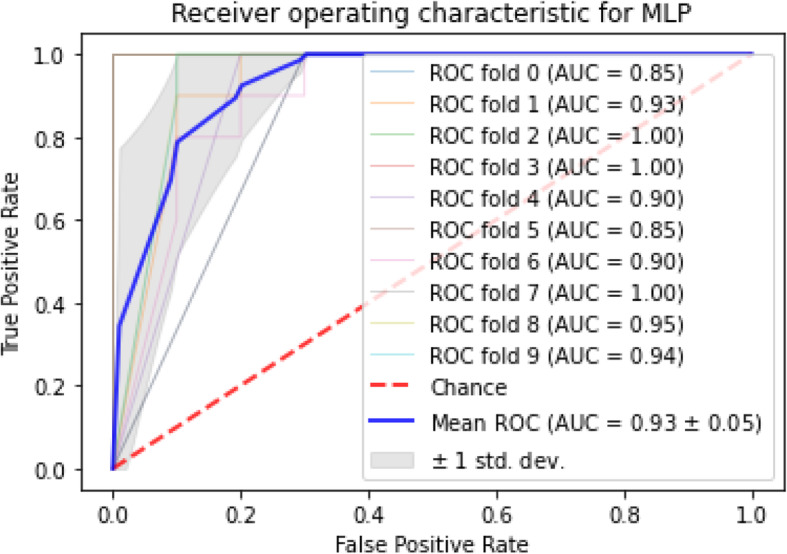
The ROC for MLP

**Table 3:** The AUC of algorithms for each iteration

Number of folds	AUC			
	KNN	SVM	ANN	Logistic Regression
1	0.85	0.93	0.85	0.99
2	0.90	0.9	0.93	0.90
3	1.00	1.00	1.00	1.00
4	0.99	1.00	1.00	1.00
5	0.91	0.98	0.90	0.95
6	0.91	0.89	0.85	0.90
7	0.84	0.91	0.90	0.90
8	1.00	1.00	1.00	1.00
9	0.82	0.9	0.95	0.90
10	0.88	0.99	0.94	0.99

**Fig. 5:**
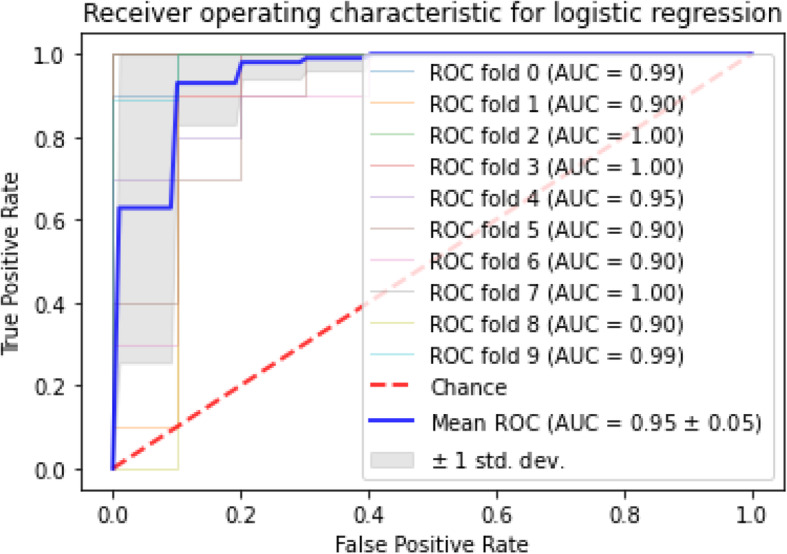
The ROC for logistic regression

AUC stands for “Area under the ROC Curve.” AUC computes the entire two-dimensional area under the whole ROC curve. According to the finding, the maximum AUC dedicated to SVM and logistic regression, among others, knn also had the highest mean AUC and minimum standard deviation of AUC scores among the approaches. The mean and standard deviation for AUC, sensitivity, and specificity of each algorithm is given in Table 4. Apart from classification AUC, sensitivity, and specificity, the Receiver Operating Characteristic (ROC) with stratified cross-validation is shown for each approach in Figs. 2, 3, 4 and 5.

**Table 4:** The mean and standard deviation of AUC, sensitivity and specificity of algorithms

Algorithm	Mean AUC + - std	Mean sensitivity+ − std	Mean specificity+ − std
KNN	0.91 + − 0.06	0.96 + − 0.06	0.85 + − 0.01
SVM	0.95 + − 0.05	0.95 + − 0.06	0.86 + − 0.01
ANN	0.93 + − 0.03	0.78 + − 0.12	0.85 + − 0.01
Logistic Regression	0.95 + − 0.05	0.92 + − 0.06	0.85 + − 0.01

ROC curves generally plot true positive rate on the Y-axis and false positive rate on the X-axis. In other words, a false positive rate of zero, and a true positive rate of one in the top left corner of the plot is called the ideal point. It means that a larger area under the curve (AUC) is usually better. According to the demonstrated ROCs, the KNN and SVM have a better area under the curve in comparison with the others.

## Discussion

For a medical diagnosis, optimized approaches to gain useful and accurate outcomes are essential. Applying machine learning and data mining methods to automate the process of diagnosis may assist practitioners to enhance the quality of their clinical decisions [32, 33].

Since T2DM is one of the prevalent diseases with severe consequences [1], developing efficient methods for early detection of the disease was the primary purpose of our research.

Regardless of high number of lncRNAs in the RNA profile of human, a few numbers of them has been proved to be biologically active. The role of the few lncRNAs has been identified but several studies discussed the significant impact of lncRNAs in diabetic people, which may represent the role of abnormal expression of lncRNAs in the incidence of T2DM [3]. According to the possible function of lncRNAs in the development of T2DM, we considered the expression levels of six lncRNAs in addition to the demographic data in 200 diabetic and healthy individuals for our study. To measure the expression of the lncRNAs we applied PBMCs which demonstrate an extensive proportion of the genes encoded in the human genome [3]. Several studies have investigated different machine learning and data mining methods to predict different diseases [15, 19, 22, 34, 35] such as heart diseases, thyroid tumors, and also diabetes type 2 diabetes prediction. In the present study, we combined four commonly used data mining algorithms (KNN, SVM, neural networks, and regression) to predict type 2 diabetes using 6 Long non-coding RNAs expression and the demographic variables for the first time, because most of the previous studies used blood test variables or the demographic data for their analysis. Receiver operating characteristic (ROC) analysis, AUC, sensitivity, and specificity measure was used to assess the diagnostic value of the six biomarkers for T2DM. The mean AUC for the KNN algorithm was obtained 91% and with 0.06 standard deviation, and we obtained the highest sensitivity (96% with the standard deviation of 0.06), among other approaches. After applying the SVM algorithm, the mean AUC obtained 95% after 10-folds with the standard deviation of 0.05, and the highest specificity, among other approaches, obtained 86% with the standard deviation of 0.01. For the ANN, we applied a multi-layer perceptron with five hidden layers, and the mean AUC of folds was 93%, and the standard deviation was 0.03. At last, for the logistic regression algorithm, our results showed 95% of mean AUC, and the standard deviation of 0.05. The lower standard deviations in the AUC scores of computed folds means the algorithm has worked with more performance [15, 17, 36]. Other studies investigated data mining algorithms for several diseases. Saravananathan and Velmurugan [21] applied several classification algorithms in their study to analyze diabetes data, including KNN. Sadri Sa’di et al. [36] compared three data mining algorithms to predict T2DM and gained 73% precision for ANN. Sidiq et al. [15]. reported about 92% accuracy for KNN and 96% accuracy for SVM algorithms applying for the Diagnosis of Various Thyroid Ailments. In another study for the heart diseases. The data mining algorithms indicated more than 70% accuracy. The investigated studies are in line with the findings of our study that these algorithms have a strong power for prediction and early detection of many diseases, including T2DM, and we obtained remarkably better accuracy for prediction, for example, the SVM and logistic regression accuracy were 95%. In our study, we also obtained a better accuracy for logistic regression that was 95% and, in comparison with other studies, is a strong point, for example, Saeidi et al. [3] conducted a study to review two Long non-coding RNA expressions in type 2 diabetes mellitus and with applying regressions reported about 65% accuracy. Another research [2] used two different Long non-coding RNA expressions in type 2 diabetes mellitus and found 81% of accuracy with the regression algorithm. In the present study, for the first time, we performed four data mining algorithms on six Long non-coding RNAs and compared their power with each other. We demonstrated that Long non-coding RNAs are effective biomarkers for data mining algorithms and have a feasible power to be applied for prediction of T2DM. Also, in this research, we optimized the parameters of every algorithm and used stratified 10-fold cross-validation to gain the best performance. To be mentioned, in the nearest neighbor’s algorithm, the parameter k was varied between one and nine to find the best-optimized method, and we selected k = 3 to have the best performance and the lowest standard deviation in the accuracy of the folds. In addition, in choosing the parameters of the artificial neural network, the number of hidden layer neurons significantly affects the accuracy of the network, so we set the parameters with two hidden layers with five and three neurons respectively to yield the best accuracy. Considering the standard deviation of scores for each algorithm, the KNN had the lowest std. Moreover, the highest accuracy among the algorithms was the SVM algorithm and Logistic regression, which had the maximum accuracy in folds, among others. We should mention that the strong points of our study are using demographic data and six Long non-coding RNAs and combining them to get the best detection power of T2DM and performing four outstanding data mining algorithms and comparing their performances. As the limitations of this study, we should account for the limited number of samples, which is due to the high costs of measuring the Long non-coding RNAs. No doubt, the higher number of samples would lead to higher performance and more reliable results.

## Conclusion

In this paper, the performance of conventional data mining classification techniques has been calculated and compared, for a dataset of patients referred for the screening of type 2 diabetes to the Shohada Hospital, Iran. The biomarker applied in this study demonstrated high diagnostic value, and the diagnostic process is suitable, which could help in the diagnosis of prediabetes and T2DM.

The classification techniques compared were support vector machine, artificial neural network, decision tree, nearest neighbors, and logistic regression. In data mining, it is not possible to say one classification technique will always work best, and it often depends on the number of samples, their distribution, and the choosing of the right algorithm. In this research work, SVM and Logistic Regression had the best Area Under Curve among methods of classification with the mean AUC of 95%. KNN and ANN also had the high mean AUC and small standard deviations of AUC scores among the approaches, KNN had the highest mean sensitivity, and the highest mean specificity belonged to SVM.

For future works, performing other data mining and machine learning methods and using higher numbers of samples are recommended to enhance the performance.

## Data Availability

The datasets used and/or analyzed during the current study are available from the corresponding author on reasonable request.
